# Gender-Related Differences in Chronic Kidney Disease-Associated Vascular Calcification Risk and Potential Risk Mediators: A Scoping Review

**DOI:** 10.3390/healthcare9080979

**Published:** 2021-08-01

**Authors:** Patrick Yihong Wu, Szu-Ying Lee, Ke-Vin Chang, Chia-Ter Chao, Jenq-Wen Huang

**Affiliations:** 1 School of Medicine, National Taiwan University College of Medicine, Taipei 100233, Taiwan; b05401019@ntu.edu.tw; 2 Nephrology Division, Department of Internal Medicine, National Taiwan University Hospital Yunlin Branch, Yunlin County 640, Taiwan; isis0525@gmail.com (S.-Y.L.); 007378@ntuh.gov.tw (J.-W.H.); 3 Department of Physical Medicine and Rehabilitation, National Taiwan University Hospital BeiHu Branch, Taipei 10845, Taiwan; kvchang011@gmail.com; 4 Graduate Institute of Toxicology, National Taiwan University College of Medicine, Taipei 100233, Taiwan; 5 Nephrology Division, Department of Internal Medicine, National Taiwan University College of Medicine, Taipei 100233, Taiwan; 6 Nephrology Division, Department of Internal Medicine, National Taiwan University Hospital BeiHu Branch, Taipei 10845, Taiwan

**Keywords:** aortic calcification, chronic kidney disease, dialysis, fetuin-A, gender, end-stage renal disease, fibroblast growth factor-23, Klotho, mineral bone disorder, osteoprotegerin, sclerostin, sex, vascular calcification, valvular calcification, vitamin D

## Abstract

Vascular calcification (VC) involves the deposition of calcium apatite in vascular intima or media. Individuals of advanced age, having diabetes mellitus or chronic kidney disease (CKD) are particularly at risk. The pathogenesis of CKD-associated VC evolves considerably. The core driver is the phenotypic change involving vascular wall constituent cells toward manifestations similar to that undergone by osteoblasts. Gender-related differences are observed regarding the expressions of osteogenesis-regulating effectors, and presumably the prevalence/risk of CKD-associated VC exhibits gender-related differences as well. Despite the wealth of data focusing on gender-related differences in the risk of atherosclerosis, few report whether gender modifies the risk of VC, especially CKD-associated cases. We systematically identified studies of CKD-associated VC or its regulators/modifiers reporting data about gender distributions, and extracted results from 167 articles. A significantly higher risk of CKD-associated VC was observed in males among the majority of original investigations. However, substantial heterogeneity exists, since multiple large-scale studies yielded neutral findings. Differences in gender-related VC risk may result from variations in VC assessment methods, the anatomical segments of interest, study sample size, and even the ethnic origins of participants. From a biological perspective, plausible mediators of gender-related VC differences include body composition discrepancies, alterations involving lipid profiles, inflammatory severity, diversities in matrix Gla protein (MGP), soluble Klotho, vitamin D, sclerostin, parathyroid hormone (PTH), fibroblast growth factor-23 (FGF-23), and osteoprotegerin levels. Based on our findings, it may be inappropriate to monotonously assume that male patients with CKD are at risk of VC compared to females, and we should consider more background in context before result interpretation.

## 1. Vascular Calcification (VC): An Introduction

The ectopic deposition of calcium-containing crystals in vascular intima or media is termed VC and involves large conduit arteries and small ones as well. VC frequently affects individuals of advanced age and those with accelerated biological ageing related to diabetes mellitus (DM) or chronic kidney disease (CKD). Findings from large population-based cohorts identified a VC prevalence of 40% to 60% and 30% to 40% among older [1,2] and middle-aged adults [3]. Meta-analyses revealed that the VC prevalence rose to 60% among patients with CKD regardless of age [4] and also increased in those with DM [5]. Calcifications affecting conduit arteries lead to vessel stiffening, increased peripheral resistance and the risk of hypertension, cardiac hypertrophy, heart failure and cardiovascular mortality. Calcifications involving microcirculation aggravate tissue perfusion through microvascular dysfunction and they induce silent ischemic episodes [6], leading to a higher risk of cardiovascular events [7,8]. VC-related adverse outcome influences become more prominent among those with CKD [9]. Valvular calcification portends a similarly worse outcome among this population [10]. VC further elevates their risk of having rapid renal function decline [11], developing morbidities including malignancies, hip fractures, pneumonia, and detrimental phenotypes like frailty [12,13]. The understanding of pathogenesis related to CKD-associated VC evolves considerably but remains elusive. It is now believed that vascular smooth muscle cells (VSMCs) and/or their neighboring constituents adopt an osteoblast-like phenotype and these trans-differentiated cells secrete osteoid matrices under pervasive exposure, leading to VC [14]. This calcification process is further augmented by redox imbalance, epigenetic changes, uremic milieu, the reduction in endogenous anti-calcific defense machineries, extracellular vesicle secretion and the dysregulated microRNA network [15,16,17,18].

## 2. Risk Factors of CKD-Associated VC: Gender Matters?

VC is one of the CKD-mineral bone disorder (CKD-MBD) components, which encompass divalent ion dysregulation (dyscalcemia, hyperphosphatemia), osteodystrophy, and ectopic calcification involving soft tissues. There are two types of VC, intimal and medial calcification. The former resides within atherosclerotic plaques based on tunica intima, and shares atherosclerotic risk factors including an advanced age, genetic susceptibility such as polymorphisms involving ApoE and Lp(a), obesity, DM and/or insulin resistance, other cardiovascular morbidities, etc. The latter type is associated with atypical risk factors such as uremic toxin exposure, osteoporosis/sarcopenia, chronic inflammation, and elevated oxidative stress/glycation endproducts [19]. Medial calcification is proposed to be more characteristic of CKD-associated VC relative to intimal calcification, although the incidence of both disease categories rises in the CKD population.

Traditional belief maintains that males have a significantly higher prevalence of atherosclerosis, are more likely to have accelerated lesion progression, be symptomatic, and to have more severe/unstable plaque features compared to females [20]. There are also studies suggesting that the influence of beneficial lifestyle changes on the risk of atherosclerosis may have gender-related differences [21]. On the contrary, females tend to occupy a greater proportion of patients with atherosclerosis or ischemic heart disease than males after 75 years [22]. Gender-related differences in atherosclerosis are mainly attributed to estrogenic action, since younger females with primary ovarian insufficiency have a significantly elevated risk of ischemic heart events and overall mortality than those without [23]. Despite the wealth of data regarding gender differences in atherosclerotic risk, few address whether gender modifies VC risk, especially CKD-associated VC. A recent study showed that males had a 3-fold higher risk of coronary artery calcification (CAC) compared to females, and the former had a more diffuse distribution of calcification than the latter [24]. Consequently, we performed a scoping literature review aiming to summarize the existing knowledge regarding gender-related differences in VC prevalence, severity, risk, and potential mediators.

## 3. Literature Search Strategy

We adopted a systematic approach to identify articles focusing on VC in patients across all stages of CKD, using the following MeSH or Emtree keywords: ‘vascular calcification’, ‘male’ or ‘female’, and ‘renal insufficiency, chronic’ or ‘renal replacement therapy’, from databases including PubMed, MEDLINE, EMBASE, Google Scholar, and Cochrane Reports between 1968 and 6 May 2021. Inclusion criteria were original investigations involving human subjects, reporting data of the gender variable and any types of VC, pulse wave velocity (PWV), serum calcification propensity (T_50_), or potential functional regulators of VC, among populations with various stages of CKD. Identified studies were initially screened by two investigators (P.Y.W. and C.T.C.) independently. This was followed by the application of exclusion criteria, consisting of review articles, those without an abstract available for screening, those that did not provide data on gender-related differences in the prevalence/course/risk of VC or its related regulators, or those that focused on non-CKD patients. We subsequently reviewed the abstract of identified articles and their reference lists to uncover additional studies containing original data of similar topics. Discrepancies between the two reviewers regarding article eligibility were resolved by arbitration from a senior investigator (J.W.H.).

We extracted relevant parameters from the identified studies, including the year and authors of publication, the stages of participants’ CKD at baseline, methods for measuring VC status and severities, the distribution of gender and/or the gender as a predictor of VC or its regulators, and results from univariate and multivariate analyses of clinical features between males and females. We organized results according to the following categories: gender-related difference in the prevalence, severities and/or courses of VC; gender as a risk factor for incident/worsening VC; and gender as a modifier for VC risk.

## 4. Summary of Findings

**Figure 1:**
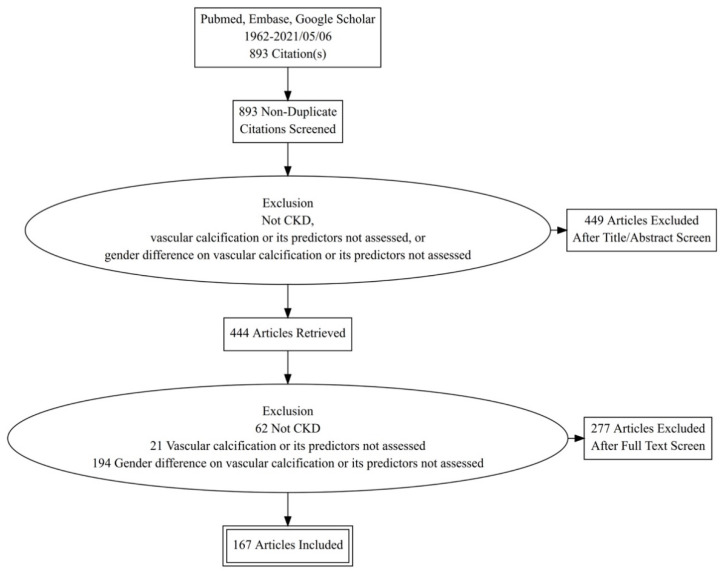
Literature selection algorithm of this review. CKD, chronic kidney disease.

A total of 893 studies were screened initially (Figure 1). After exclusion of articles that met our exclusion criteria following the title/abstract/full-text review, 167 articles were retained for summarization. In most studies of non-dialysis CKD patients, the estimated glomerular filtration rate (eGFR) was obtained based on the Modification of Diet in Renal Disease (MDRD) formula, while some earlier studies used elevated serum creatinine to identify those with CKD. Staging of CKD, when available, was done according to the Kidney Disease Improving Global Outcome (KDIGO) classification [25]. For the estimation of VC risk in multivariate analyses, factors that were adjusted for included at least age and gender and could further include interfering parameters such as body size, comorbidities, medications, and/or laboratory indicators.

### 4.1. Gender-Related Differences in the Prevalence and/or Severity of CKD-Associated VC

In total, 101 (60.5%) original investigations evaluated the differences in the prevalence and/or severity of CKD-associated VC between different genders [26,27,28,29,30,31,32,33,34,35,36,37,38,39,40,41,42,43,44,45,46,47,48,49,50,51,52,53,54,55,56,57,58,59,60,61,62,63,64,65,66,67,68,69,70,71,72,73,74,75,76,77,78,79,80,81,82,83,84,85,86,87,88,89,90,91,92,93,94,95,96,97,98,99,100,101,102,103,104,105,106,107,108,109,110,111,112,113,114,115,116,117,118,119,120,121,122,123,124,125,126]. Among these articles, 27 (26.7%), 64 (63.4%) and 10 (9.9%) included patients with non-dialysis CKD, dialysis-dependent CKD, and renal transplant recipients, respectively (Supplementary Table S1). In those involving patients with non-dialysis CKD, one-third (n = 9) found no differences in VC prevalence/risk between genders, while two-thirds (n = 18) reported that males had a higher VC prevalence/severity than females. Interestingly, in reports involving patients with non-dialysis CKD, those directly assessing VC based on coronary computed tomography (CT) or plain radiography for aortas tended to derive results that suggested higher male VC prevalence/severity, when the case number was large. On the other hand, studies that involved the assessment of PWV or T_50_ tended to have neutral results (Supplementary Table S1).

In studies involving patients with dialysis-dependent CKD, 37 (57.8%) found no differences in VC prevalence/severity between genders, while 22 (34.4%) found that males had a higher VC prevalence than females (Supplementary Table S1). On the contrary, 5 (7.8%) reports identified that females had a higher VC prevalence than males. If coronary VC was the phenotype of interest in studies involving patients with dialysis-dependent CKD, males were more likely to have a higher prevalence/severity of VC than females (neutral vs. male higher, 5 out of 37 (13.5%) vs. 8 out of 22 (36.4%) studies using Agatston scores as surrogates). Reports that identified a higher VC prevalence/severity in dialysis-dependent CKD females than males tended to originate from Asian countries and involved carotid artery calcification (Supplementary Table S1).

In studies involving patients receiving renal transplant (CKD stage 5T), 6 (60%) found no differences in VC prevalence/severity between genders, while 4 (40%) reported more males with a higher VC prevalence/severity than females (Supplementary Table S1). Similar to studies involving dialysis-dependent CKD patients, if coronary VC was the phenotype of interest, males were more likely to be reported as having higher prevalence/severity of VC than females (neutral vs. male higher, 1 out of 6 (16.7%) vs. 2 out of 4 (50%) studies using Agatston scores as surrogates).

Few studies adopted a histopathological approach to identify gender-related differences in CKD-associated VC prevalence/severity (n = 5; 5.0%), and even fewer addressed gender-related differences in valvular calcification (n = 3, 3.0%) (Supplementary Table S1). There were reports suggesting neutral results or higher male prevalence/severity of VC using the histopathological approach, while none of the reports addressing valvular calcification identified gender-related differences in prevalence.

### 4.2. Gender-Related Differences in the Adjusted Risk of CKD-Associated VC Presence or Severity

**Figure 2:**
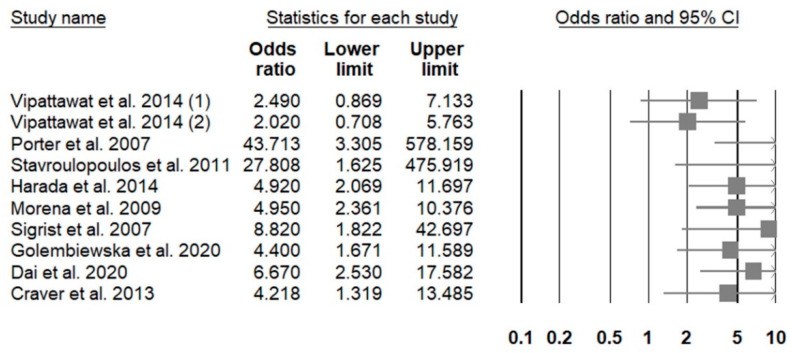
A forest plot showing the male to female risk among patients with non-dialysis chronic kidney disease.

**Figure 3:**
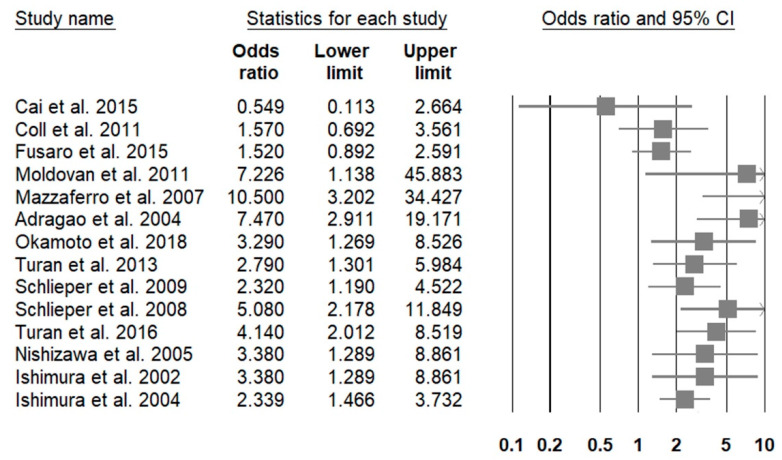
A forest plot showing the male to female risk among patients with dialysis-dependent chronic kidney disease.

A total of 62 (37.1%) original investigations reported gender-associated adjusted risk of having higher prevalence/severity of VC among patients with CKD [40,42,44,47,48,58,63,64,65,67,68,84,86,88,90,93,95,100,101,102,106,112,113,116,117,119,120,121,122,123,124,125,126,127,128,129,130,131,132,133,134,135,136,137,138,139,140,141,142,143,144,145,146,147,148,149,150,151,152,153,154,155]. Among these articles, 17 (27.4%), 40 (64.5%) and 5 (8.1%) included patients with non-dialysis CKD, dialysis-dependent CKD, and renal transplant recipients, respectively (Supplementary Table S2). In those involving patients with non-dialysis CKD, only 3 (17.6%) found no differences in VC risk between genders, while 14 (82.4%) reported that males had a higher adjusted risk of VC than females. A forest plot illustrating the risk of VC presence or greater severity associated with male gender in those with non-dialysis CKD is shown in Figure 2. Among studies enrolling more than 100 patients, most concluded that males exhibited a significantly higher risk of having VC involving coronary arteries, aortas or higher PWVs than females (Supplementary Table S2). The male-to-female odds ratio (ORs) of developing VC ranged between 4.2 and 43, depending on the cohort size and the combinations of adjusted variables, regardless of assessment methods for VC. In those involving patients with dialysis-dependent CKD, relatively few (n = 13; 32.5%) found no gender-related alterations in VC risk, and reports identifying a higher VC risk among males (n = 25; 62.5%) significantly outnumbered those identifying a higher VC risk among females (n = 2; 5%) (Supplementary Table S2). A forest plot illustrating the risk of VC presence or greater severity associated with male gender in those with dialysis-dependent CKD is shown in Figure 3. The male-to-female ORs or relative risks (RRs) of CKD-associated VC were between 1.9 and 10.5. It appears that the male-to-female OR/RRs for VC tend to be lower if aortic calcification was considered (between 2.2 and 3.3) and higher if CAC was studied (between 2.8 and 10.5) (Supplementary Table S2). Reports identifying a higher CKD-associated VC risk among females than males focused on aortic calcification and carotid artery calcification. Among studies involving patients receiving renal transplantation, most (n = 4; 80%) concluded that males had a significantly higher risk of VC than females, and 75% of them assess CAC (Supplementary Table S2).

### 4.3. Potential Mediators of Gender-Related Differences in CKD-Associated VC Risk

A total of 53 original investigations addressed potential modifiers of the gender-VC relationship among patients with CKD, based on our literature summary [36,40,43,58,59,61,66,86,106,107,110,115,129,130,134,152,156,157,158,159,160,161,162,163,164,165,166,167,168,169,170,171,172,173,174,175,176,177,178,179,180,181,182,183,184,185,186,187,188,189,190,191,192]. We divided these mediators into 7 categories: genetic susceptibility, body composition and nutrition/inflammation status, medication, epigenetic mediators, divalent ion/electrolyte balances, osteogenesis regulators, and miscellaneous (Table 1). Studies involving polymorphisms of the vitamin K metabolism-related gene (VKORC1) and calcification inhibitors (matrix Gla protein; MGP) did not find gender-related differences in allelic distributions. In terms of body composition, males had significantly higher pericardial fat areas, low density lipoprotein (LDL) cholesterol, and angiotensin-converting enzyme 2 (ACE2) levels but lower total fat mass, adiponectin, and high density lipoprotein (HDL) cholesterol than females (Table 1). Fluid status, either in the form of extracellular fluid volume or serum copeptin levels, did not exhibit gender-related differences when CKD-associated VC risk was considered. There were studies addressing gender-related differences in CKD-associated VC risk modifying medication (statin) or microRNA levels, but the results were all neutral. Divalent ions and electrolytes such as serum phosphorus, calcium, and magnesium were reported to influence the risk of CKD-associated VC [193]; in our literature summary, females were found to have a higher probability of hyperphosphatemia than males (Table 1), but there was no difference in serum magnesium or calciuria levels. Thirty (56.6%) reports intended to examine gender-related differences in multiple osteogenesis regulator levels, including MGP (n = 2; 6.7%), fetuin-A (n = 4; 13.3%), fibroblast growth factor-23 (FGF-23) (n = 2; 6.7%), osteoprotegerin (n = 8; 26.7%), parathyroid hormone (PTH) (n = 2; 6.7%), sclerostin (n = 8; 26.7%), soluble Klotho (n = 3; 10%), and vitamin D (n = 2; 6.7%). No gender-related differences were observed regarding fetuin-A, while most studies concluded that males had significantly lower dephosphorylated uncarboxylated MGP and Klotho but higher sclerostin and vitamin D levels than females (Table 1). Studies focusing on gender-related differences in PTH, FGF-23, and osteoprotegerin levels among patients with CKD had controversial findings; those addressing PTH or FGF-23 had either neutral results or higher levels in females than males. Studies addressing osteoprotegerin yielded neutral, male higher, or female higher results. Interestingly, reports garnering higher patient numbers uniformly concluded that females had higher osteoprotegerin levels than males (Table 1). Finally, several studies focusing on VC identified gender-related differences in signal peptide-CUB-EGF domain-containing protein 1 (SCUBE1) and tissue advanced glycation (Table 1), which might bear potential influences on gender-related VC risk.

## 5. Overall Interpretations

In this literature review involving 167 studies of CKD-associated VC, we found that the majority identified males with a higher VC prevalence/severity compared to females, although stages of CKD, the approaches used to measure VC, and the number of cases per study may account for the heterogeneity in study findings. In patients with non-dialysis CKD, males might not necessarily have a higher VC prevalence/severity, especially when studies assessed VC surrogates, except for those focused on CAC. In patients with dialysis-dependent CKD, males still exhibited a higher VC risk, but heterogeneity persisted. Females from Asian countries exhibited a higher tendency of having carotid artery calcification relative to males. These findings appeared to be more uniform after multivariable adjustment, as studies in patients with non-dialysis dependent and dialysis-dependent CKD mostly supported a higher VC risk among males. Potential contributors to the observed gender-related differences can be multiple, including pericardial/total fat, lipid profile, inflammatory status, variations in MGP, soluble Klotho, vitamin D, sclerostin, PTH, FGF-23, and osteoprotegerin levels.

### 5.1. Intimal or Non-Intimal Calcification: A Potential Consideration for Gender-Related Differences in CKD-Associated VC Risk

Findings from our literature summary suggest that reports focusing on CAC more frequently identified a male preponderance in VC prevalence/risk (Supplementary Tables S1 and S2). Coronary atherosclerosis frequently begins with the retention of LDL and/or Lp(a) in subendothelial extracellular matrix, leading to local inflammation, cellular necrosis, and attraction of monocytes with phagocytosis of the degraded content. The necrotic core serves as the niche of calcification nucleation, assisted further by the enhanced release of pro-calcifying vesicles and the reduced expression of mineralization inhibitors by neighboring cells [194]. CAC is therefore deemed more akin to a symbol of atherosclerotic intimal calcification, although medial calcification exists simultaneously. On the other hand, calcifications involving smaller diameter vessels such as arteriovenous accesses, or digital arteries are more likely to be medially located and are pathophysiologically distinct from intimal type calcification [195]. Since male gender correlates with a higher risk of atherosclerosis and potentially atherosclerotic calcification [20], studies focusing on CAC may be inclined to derive the finding that males have an increased VC risk relative to females.

### 5.2. Reports Favoring a Higher Risk of CKD-Associated VC in Males: Plausible Mediators

We discovered that in CKD patients, especially those with non-dialysis status, males were at a higher risk of VC compared to females (Supplementary Table S2). It was further disclosed that males had a significantly higher proportion of pericardial fat and higher levels of serum ACE2, LDL, vitamin D, and sclerostin than females, but lower HDL, adiponectin, uncarboxylated MGP and soluble Klotho (Table 1). Prior studies showed that a higher amount of pericardial fat tissues was associated with a greater CAC severity at baseline and a higher speed of CAC progression, through their paracrine and endocrine effects of inducing inflammation, oxidative stress, and endothelial dysfunction [196]. A study disclosed that aortic ACE2 expression was significantly up-regulated in calcified aortas [197], and ACE2 presumably plays an important role in perpetuating local inflammation. Calcitriol supplementations have been reported to intensify the severity and accelerate the tempo of VC in models of CKD and DM [198,199], suggesting that differences in vitamin D levels may be partially responsible for gender-related differences in VC risk. Sclerostin, an inhibitor of the canonical Wnt/β-catenin pathway originally thought to be solely expressed in bones, is found to be up-regulated in VSMCs and serves as a predisposing factor for VC [200]. It is likely that higher expressions of sclerostin in male CKD patients contribute to their tendency of developing VC. Along the same line, anti-calcific factors in CKD such as MGP and Klotho [201,202] were also lower in male CKD patients (Table 1), further paving the ground for their pro-calcific tendency. Dyslipidemia, in the forms of high LDL or low HDL, is associated with an increased risk of VC progression through their pathogenic linkage to atherosclerosis and local inflammation [203,204]. From this perspective, male CKD patients may harbor several risk features that predispose themselves to VC development.

### 5.3. Reports Favoring Higher Risk of CKD-Associated VC in Females: Clinical Implications and Plausible Mediators

Few studies (5% to 7%) in our summary identified females as having an increased VC risk relative to males (Supplementary Tables S1 and S2), while they predominantly focused on aortic or carotid artery calcification and tended to originate from Asian countries. Experimental reports showed that through binding to estrogen receptor α, estrogen could inhibit receptor activator of nuclear factor κ ligand (RANKL) signaling, leading to VSMC trans-differentiation and calcification [205]. However, there are also studies suggesting that estrogen analogs could aggravate VSMC calcification [206]. These findings indicate that the influence of estrogen on VC can be complex and scenario-dependent, unlike that exerted by testosterone, which is consistently pro-calcific [207]. Several other features can further modify these gender-related differences in VC risk, including the higher serum phosphate, FGF-23, and PTH levels observed in female CKD patients (Table 1). Elevated levels of FGF-23 and PTH parallel the worsening of CKD-associated VC in clinical studies, and participate in VC pathogenesis according to experimental reports [208]. Consequently, alterations in these hormones may be responsible for the increased risk of CKD-associated VC in females, at least partially.

### 5.4. Gender-Related Differences in Osteoprotegerin Levels and CKD-Associated VC

We observed substantial heterogeneity in studies reporting gender-related differences in serum osteoprotegerin levels focusing on VC; there were reports yielding no gender-related difference, while others reported either higher levels in males or in females (Table 1). It is clear that studies enrolling more patient numbers (>300) tend to identify higher osteoprotegerin levels in females but not males with CKD. In vitro studies indicated that estrogen could stimulate the up-regulation of osteoprotegerin in osteoblasts, but the direction of effect may depend on the types of estrogen receptors [209]. Consequently, clinical findings that higher serum osteoprotegerin levels alternated between different genders are not unexpected, and a careful interpretation of results in the context of sample size and other influencing factors is needed.

## 6. Conclusions

Gender-related differences in various biological phenotypes do exist, and such differences greatly affect cardiovascular risk estimation and carry public health importance. The male gender is traditionally regarded as a risk trait for atherosclerosis, but whether this relationship applies to VC remains debatable. VC encompasses a complex molecular interplay between endothelial cells, VSMCs, adventitial fibroblasts, pericytes, and even infiltrating macrophages, further compounded by the influences of uremic milieu and pro-/anti-calcific factors. We systematically identified existing reports of CKD-associated VC or its potential regulators containing data about gender distributions, and synthesizing their messages. Though a higher risk of CKD-associated VC risk was observed in males among the majority of original investigations, heterogeneity in findings were found; multiple large-scale studies yielded neutral findings, and few reported a higher risk of CKD-associated VC among females. Clinically speaking, differences in findings of gender-related VC risk may result from variations in outcome assessment approaches, the anatomical segments of interest, sample size, and even ethnic origins of participants. Biologically speaking, plausible mediators of such gender-related difference may include body composition changes, lipid profiles, inflammatory status, and diversities in MGP, soluble Klotho, vitamin D, sclerostin, PTH, FGF-23, and even osteoprotegerin levels. Based on our findings, it may not be appropriate to assign a high-risk VC category to males with CKD, and more background in context should be contemplated before reaching conclusions.

## Figures and Tables

**Table 1:** Potential gender-related modifiers of vascular calcification in existing studies.

Authors	Country	Time	CKD Stages	Sample Size	Gender Effect	Data	Potential Modifiers	Ref.
Genetic susceptibility								
Holden et al.	Canada	2014	3–5	167	Neutral	CC vs. CG/GG, male 68% vs. 55%, *p* = 0.11	VKORC1 (vitamin K epoxide reductase complex 1) polymorphism	[166]
Yoshikawa et al.	Japan	2013	5D (HD)	134	Neutral	CC vs. CT vs. TT genotype, male 14% vs. 48% vs. 33%, *p* = 0.53	MGP Genotype T-138C	[110]
Body composition and inflammatory status								
Axelsson et al.	Sweden	2008	5	198	Male lower	Male β = −1.68, *p* < 0.001	Total fat mass	[157]
Park et al.	Korean	2018	1–5	1741	Neutral	Quartile 1 (lowest) vs. 2 vs. 3 vs. 4, male 80.6% vs. 59.0% vs. 39.7% vs. 39.6, *p* < 0.001 Male β = 0.095 (−0.421~0.231), *p* = 0.566	ECF excess	[179]
Choi et al.	Korea	2019	5D (HD)	97	Neutral	Marker count 2 vs. 1 vs. 0, male 61.5% vs. 41.9% vs. 34.1%, *p* = 0.216	Nutrition (albumin) and inflammation (CRP) markers	[43]
Golembiewska et al.	Sweden	2020	5	149	Neutral	High vs. middle vs. low levels, male 71% vs. 67% vs. 65%, *p* = 0.84 Regression model, male β −0.08, *p* = 0.31	Copeptin	[134]
Harada et al.	Brazil	2014	2–5	117	Male more severe	Area in males vs. females, 220.4 ± 110.5 vs. 135.4 ± 76.0, *p* < 0.001	Pericardial fat	[58]
Turan et al.	Turkey	2013	5D (HD)	191	Neutral	Low vs. middle vs. high, female 46% vs. 39% vs. 58%, *p* = 0.1	Epicardial fat	[152]
Miyatake et al.	Japan	2020	5T	50	Female higher HDL, adiponectin but lower LDL	For LDL-C, male vs. female 113.0 (97.0–132.5) vs. 90.0 (76.5–98.3), *p* < 0.01 For HDL-C, male vs. female 57.0 (51.0–67.0) vs. 78.0 (66.8–96.5), *p* < 0.01 For HMW-ADPN, male vs. female 2.48 (1.62–3.33) vs. 4.52 (3.02–6.79), *p* < 0.01 For LMW-ADPN, male vs. female 1.67 (1.14–1.89) vs. 2.26 (1.85–2.83), *p* < 0.01 For CTRP9, male vs. female 2.08 (2.01–2.13) vs. 2.03 (2.00–2.07), *p* > 0.05	Lipid profile, circulating C1q/TNF-a related protein-9 (CTRP9), and adiponectin	[86]
Zhang et al.	China	2015	5D (HD)	90	Male higher	≤30 vs. 30–60 vs. ≥ 60 U/L, male 29.4% vs. 46.1% vs. 85.7%, *p* < 0.001 Male correlation coefficient 0.362, *p* < 0.001	Serum ACE2	[190]
Medications								
Chen et al.	Sweden	2017	5D, 5T	240	Neutral	Users vs. non-users, male 65% vs. 62%, *p* = 0.678	Statin use	[41]
Epigenetic mediators								
Chao et al.	Taiwan	2019	5D	223	Neutral	High vs. low levels, female 63% vs. 51%, *p* = 0.14	microRNA-125b	[160]
Divalent ion/electrolyte balance								
Block et al.	United States	1998	5D (HD)	6407	Female higher	Male OR 0.774, *p* = 0.0001	Serum phosphorus > 6.5 mg/dL	[158]
Zou et al.	China	2016	1–5	296	Neutral	Tertile 1 (lowest) vs. 2 vs. 3, male 60.2% vs. 50.0% vs. 61.2%, *p* = 0.21	Serum phosphorus	[192]
Karsli Ceppioğlu et al.	Turkey	2011	3–5	84	Female higher	Female vs. male, higher in the former, *p* = 0.002	Serum copper	[170]
Ikee et al.	Japan	2016	5D (HD)	86	Neutral	Male vs. female, 2.51 ± 0.38 vs. 2.42 ± 0.33, *p* = 0.52	Serum magnesium	[167]
Stolic et al.	Serbia	2016	5D (HD)	88	Neutral	Male vs. female, mean 1.2 (0.7–1.6) vs. 1.2 (0.8–1.5) mmol/L, *p* = 0.896	Serum magnesium	[185]
Ramalho et al.	Brazil	2019	3–4	356	Neutral	Tertile 1 (lowest) vs. 2 vs. 3, 59% vs. 64% vs.66%, *p* = 0.5 Male β = 0.22 (−0.02~0.45), *p* = 0.07	Urinary calcium excretion	[180]
Osteogenesis/mineralization regulators								
Aoun et al.	Lebanon	2017	5D (HD)	50	Male lower	<5000 vs. >5000 pmol/L, male 68.3% vs. 22.2%, *p* = 0.02	Dp-ucMGP	[156]
Schlieper et al.	Serbia	2011	5	188	Neutral	<6139 vs. >6139 pmol/L, female OR 0.62 (0.35–1.11), *p* = 0.11	dp-cMGP	[182]
Okamoto et al.	Japan	2018	5D (HD)	230	Neutral	<0.213 vs. ≥0.213 g/L, male 61% vs. 68%, *p* = 0.400 Male OR 0.48 (0.22–1.02), *p* = 0.056	Fetuin-A	[178]
Chen et al.	Taiwan	2013	5D (HD)	238	Neutral	Tertile 1 (lowest) vs. 2 vs. 3, female 55% vs. 48% vs. 57%, *p* = 0.5	Fetuin-A	[161]
Stenvinkel et al.	Sweden	2005	5	258	Neutral	Male vs. female, median 0.225 vs. 0.223 g/L, *p* > 0.05	Fetuin-A	[184]
Metry et al.	Sweden	2008	5D (HD)	222	Neutral	High Fetuin-A/Low CRP vs. High Fetuin-A/High CRP vs. Low Fetuin-A/Low CRP vs. Low Fetuin-A/High CRP, 60.9% vs. 52.5% vs. 44.4% vs. 58.8%, *p* > 0.05	Fetuin-A and CRP	[174]
Turan et al.	Turkey	2016	5D (HD)	224	Neutral	<35.5 vs. 35.5–123.4 vs. >123.4 pg/mL, male 56.6% vs. 46.5% vs. 50.0%, *p* = 0.38	FGF-23	[106]
Nishiura et al.	Japan	2009	5D (HD)	99	Male higher	Low vs. high, male 56% vs. 75.5%, *p* = 0.057 Male HR 3.034 (1.028–8.948), *p* = 0.044	Osteoprotegerin	[177]
Hou et al.	Taiwan	2019	5D (HD)	120	Female lower	72.97–237.13 vs. 237.36–323.54 vs. 329.61–835.78 pg/L, female 62.5% vs. 55.0% vs. 32.5%, *p* = 0.008	Osteoprotegerin	[61]
Sigrist et al.	United Kingdom	2009	3–4	134	Neutral	≤25 vs. >25 pmol/L, male 63% vs. 70%, *p* = 0.47	Osteoprotegerin	[183]
Nakashima et al.	Japan	2011	5D (HD)	151	Neutral	Male β = 0.531, *p* = 0.51	Osteoprotegerin	[175]
Scialla et al.	United States	2011	1–5	351	Female higher	1.21–5.03 vs. 5.05–7.45 vs. 7.46–22.31 pmol/L, female 24.8% vs. 26.5% vs. 40.2%, *p* = 0.02 Female related OPG % difference 10.2%, *p* = 0.05	Osteoprotegerin	[181]
Gupta et al.	Hungary	2021	5D (HD)	982	Female higher	<3.2 vs. 3.2–4.39 vs. >4.39 pmol/L, male 63% vs. 57% vs. 52%, *p* = 0.015	Osteoprotegerin	[165]
Nemeth et al.	Hungary	2015	5T	993	Male lower	<3.2 vs. 3.2–4.39 vs. >4.39 pmol/L, male 63% vs. 58% vs. 52%, *p* = 0.02	Osteoprotegerin	[176]
Chae et al.	Korea	2018	1–5	1832	Neutral	Quartile 1 (lowest) vs. 2 vs. 3 vs. 4, female 36.2% vs. 43.4% vs. 42.5% vs. 38.6%, *p* = 0.517 (mean, 6.76 pmol/L)	Osteoprotegerin	[115]
Jean et al.	France	2011	5D (HD)	1138	Neutral	<50 vs. ≥50 pg/mL, female 43% vs. 40%, *p* > 0.05	Parathyroid hormone	[169]
González-Parra E et al.	Spain	2015	1–5	704	Female higher in both	For PTH, male r = −0.084 (−0.155~−0.012), *p* = 0.0215 For FGF-23, male r = −0.191 (−0.301~−0.080), *p* = 0.0007	Parathyroid hormone and FGF-23	[164]
Thambiah et al.	United Kingdom	2012	3B-4	77	Male higher	Male vs. female, mean 50.5 vs. 34 pmol/L, *p* = 0.015 Male β = 0.23, p = 0.024	Sclerostin	[186]
Kuo et al.	Taiwan	2019	5D (PD)	89	Male higher	<80.2 vs. ≥80.2 pmol/L, male 36.4% vs. 62.2%, *p* = 0.015 Male OR 2.882 (1.219–6.815), p = 0.016	Sclerostin	[172]
Goncalves et al.	Brazil	2014	5D (HD)	91	Neutral	High vs. low levels, male 68.9% vs. 52.2%, *p* = 0.103 Regression model, male OR 0.82 (0.39–1.75), *p* = 0.62	Sclerostin	[163]
Viaene et al.	Belgium	2013	5D (HD)	100	Neutral	Low vs. high, female 47% vs. 35%, *p* = 0.2	Sclerostin	[188]
Claes et al.	Belgium	2013	1–5	154	Male higher	Male gender significantly associated with higher levels (*p* = 0.006)	Sclerostin	[162]
Zou et al.	China	2020	5D	165	Male higher	Among PD patients, male β = 0.259 (0.052–0.416), *p* = 0.012	Sclerostin	[191]
Jean et al.	France	2016	5D (HD)	227	Female lower	Female OR 0.16 (0.075−0.362)	Sclerostin	[66]
Evenpoel et al.	Belgium	2015	5T	268	Male higher	Male gender significantly associated with higher levels (*p* = 0.002)	Sclerostin	[130]
Zhang et al.	China	2019	5D (HD)	105	Neutral	Quartile 1 (lowest) vs. 2 vs. 3 vs. 4, male 57.7% vs.48.1% vs. 46.2% vs. 65.4%, *p* = 0.473	Soluble Klotho	[189]
Buiten MS	Netherlands	2014	5D	127	Female higher	<460 vs. >460 pg/mL, female 16% vs. 31%, *p* < 0.05	Soluble Klotho	[159]
Cai et al.	China	2015	5D (HD)	129	Neutral	≤379 vs. 379–613 vs. 613–817 vs. >817 pg/mL, male 59.4% vs. 54.5% vs. 56.3% vs. 53.1%, *p* > 0.05	Soluble Klotho	[129]
Jean et al.	France	2008	5D (HD)	253	Female lower levels	Deficient vs. sufficient, female 53% vs. 28%, *p* < 0.05	Vitamin D (25-OH-D) deficiency	[168]
Chang et al.	Republic of Korea	2012	5D (HD)	289	Female lower levels	Female OR 3.892 (1.678–9.025), *p* = 0.002	Vitamin D (25-OH-D) deficiency (<15 ng/mL)	[36]
Miscellaneous								
Kato et al.	Japan	2009	5D (HD)	68	Neutral	No correlation with gender	Serum pre-B cell colony-enhancing factor/visfatin	[171]
He et al.	China	2018	5D (HD)	150	Neutral	Male correlation coefficient 0.106, *p* = 0.114	Serum irisin	[59]
Liabeuf et al.	France	2013	2–5, 5D	139	Neutral	≤0.041 vs. >0.041 mg/dL, male 63% vs. 57%, *p* = 0.5	Free p-cresylglucuronide	[173]
Ulusoy et al.	Turkey	2012	5D (HD)	103	Male higher	Pre-dialysis male vs. female levels, 314.7 ± 157.1 vs. 210.4 ± 115.4 ng/mL, *p* < 0.001	Signal peptide-CUB-EGF domain-containing protein 1 (SCUBE1)	[187]
Wang et al.	Hong Kong	2014	3–5	300	Male lower	Male correlation coefficient −0.14, *p* = 0.02	Tissue AGEs (surrogated by skin autofluorescence)	[107]

ADPN, adiponectin; AGE, advanced glycation endproduct; CRP, C-reactive protein; Dp-ucMGP, dephosphorylated-uncarboxylated Matrix Gla protein; ECF, extracellular fluid; FGF-23, fibroblast growth factor-23; HD, hemodialysis; HDL, high density lipoprotein; HMW, high molecular weight; HR, hazard ratio; LDL, low density lipoprotein; LMW, low molecular weight; MGP, matrix Gla protein; OR, odds ratio; PD, peritoneal dialysis.

## Data Availability

The raw data for conducting this analysis are available upon reasonable request to the corresponding author and the first author.

## Supplementary Materials

The following are available online at https://www.mdpi.com/article/10.3390/healthcare9080979/s1, Table S1: Gender-related differences in the prevalence and/or severity of vascular calcification in existing studies, Table S2: Gender-related adjusted risk of vascular calcification in existing studies.
