# Supplementary material for: Gender-Related Differences in Chronic Kidney Disease-Associated Vascular Calcification Risk and Potential Risk Mediators: A Scoping Review
Source: Healthcare (Basel). 2021 Aug 1;9(8):979. doi: 10.3390/healthcare9080979 (PMC8394860; doi:10.3390/healthcare9080979)
Supplement: Supplementary file 1 [file healthcare-09-00979-s001.zip › healthcare-1287432-supplementary.pdf]

Supplementary Table S1. Sex-related differences in the prevalence and/or severity of vascular calcification.

Authors	Country	Time	CKD stages	Sample size	Findings	Data	Calcification assessment method	Ref
Non-dialysis CKD								
Jansson *et al*	Sweden	2019	3-4	84	Neutral	With vs. without, male 79% vs. 67%, p = 0.351	Abdominal aortic calcification on computed tomography	[65]
Etta *et al.*	India	2017	4-5	95	Neutral	With vs. without, 70% vs. 71.8%, p = 0.58	Lumbar spine lateral radiography	[54]
Mizuiri *et al.*	Japan	2018	2-5	145	Neutral	Coronary calcification quartile 4 vs. 3 vs. 2 vs. 1, male 62.9% vs. 64.9% vs. 54.1% vs. 63.9%, $p > 0.05$<br>Iliac calcification quartile 4 vs. 3 vs. 2 vs. 4, male 58.3% vs. 65.8% vs. 68.6% vs. 52.8%, $p > 0.05$	Coronary artery calcification (Agatston score) and common iliac artery calcification by computed tomography	[87]
Fayed *et al.*	Egypt	2019	5	172	Neutral	Intimal vs. medial vs. none, male 37.9% vs. 64.9% vs. 58.1%	Arterial wall calcification based on intraoperative arterial biopsy	[56]
Chiu *et al*	United States	2010	Proteinuric (1-5)	225	Neutral	Group 4 (severe) vs. 3 vs. 2 vs. 1, male 61% vs. 64% vs. 47% vs. 45%, p = 0.09	Coronary artery calcification (Agatston score)	[42]
Bundy *et al*	United States	2019	2-4	1274	Neutral	Quartiles 4 vs. 3 vs. 2 vs. 1, female 46% vs. 45% vs. 49% vs. 47%	T_{50} (propensity of calcification)	[114]
Chae *et al.*	Korea	2018	1-5	1832	Neutral	Quartile 4 vs. 3 vs. 2 vs. 1, female 38.1% vs. 37.0% vs. 41.3% vs. 44.3%, p = 0.101	Pulse wave velocity	[115]
Abd Alamir *et al.*	United States	2015	2-3	2070	Neutral	With vs. without, male 49.6% vs. 54.5%, p = 0.1	Mitral annular calcification by coronary computed tomography	[112]
Bundy *et al.*	United States (CRIC)	2019	2-4	3404	Neutral	Quartile 1 (severe) vs. 2 vs. 3 vs. 4, female 47% vs. 48% vs. 44% vs. 41%, $p > 0.05$	T_{50} (propensity of calcification)	[34]

Merjanian *et al.*	United States	2003	3-5	32	Male more severe	Male vs female, scores 619 vs. 232, $p < 0.001$	Coronary artery calcification (Agatston score)	[85]
Di Iorio *et al.*	Italy	2006	4-5, 5D	44	Male more severe	Scores >400 vs.<400, male 75% vs. 42%, $p = 0.05$	Coronary artery calcification (Agatston score)	[51]
Tomiyama *et al.*	Brazil	2010	2-4	50	Male more common	With vs. without, male 79% vs. 47%, $p = 0.02$	Coronary artery calcification (Agatston score)	[105]
Porter *et al.*	United Kingdom	2007	3-4	112	Male more common	In non-diabetic group, VC prevalence in male vs. female, 60% vs. 26%, $p = 0.003$	Coronary artery calcification (Agatston score)	[124]
Harada *et al*	Brazil	2014	2-5	117	Male more severe	Score >0 vs. score =0, male 78.7% vs. 42.9%, $p < 0.001$	Coronary artery calcification (Agatston score)	[58]
Chue *et al.*	United Kingdom	2012	3	120	Male more common	Male vs. Female, 67% vs. 43%, $p = 0.01$	Lumbar spine lateral radiography	[44]
Morena *et al*	France	2009	1-5	133	Male more common	Severe vs. minor, 73.6% vs. 36.1%, $p < 0.0001$	Coronary artery calcification (Agatston score)	[88]
Sigrist *et al.*	United Kingdom	2006	4-5D	134	Male more severe	Tertiles 3 vs. 2 vs. 1, male 81% vs. 71% vs. 46%, $p < 0.001$	Superficial femoral artery calcification by computed tomography	[102]
Jiménez Villodres *et al.*	Spain	2018	3	139	Male more common	With and without abdominal aortic calcification, male 80% vs. 63%, $p < 0.05$ With and without KI, male 69% vs. 72%, $p > 0.05$	Lateral lumbar radiography (Kauppila score) and abdominal aortic calcification on computed tomography	[69]
Zhou *et al.*	Sweden	2018	1-5	151	Male more common	Male vs. female: 76% vs. 69%, $p < 0.05$	Lateral lumbar radiography (Kauppila score)	[111]

Dai *et al*	Sweden	2020	5	152	Male more severe	Moderate to extensive vs. no to minimal, male 81% vs. 56%, $p = 0.002$	Histopathology of epigastric artery calcification	[116]
Craver *et al*	Spain	2013	3-4	178	Male more severe	Scores >5 vs. 1-5 vs. 0, male 83% vs. 80% vs. 70%, $p = 0.017$	Lateral lumbar radiography (Kauppila score)	[48]
Lioufas *et al.*	Multicenter (IMPROVE-CKD)	2020	3b-4	278	Male more common	With vs. without, male 73% vs. 55%, $p = 0.02$	Abdominal aortic calcification by computed tomography	[79]
Wang *et al.*	Hong Kong	2014	3-5	300	Male more severe	Scores ≥400 vs. 100–399 vs. 1–99 vs. 0, male 77.3% vs. 56.9% vs. 57.5% vs. 38.7%, $p < 0.001$	Coronary artery calcification (Agatston score)	[107]
Kestenbaum *et al.*	United States	2009	3-5	562	Male more common	With vs. without, male 45% vs. 29%, $p < 0.001$	Coronary artery calcification (Agatston score)	[119]
Lee *et al.*	Korea	2006	2-5	1078	Male more common	With vs. without, male 73.7% vs. 54.4%, $p < 0.001$	Coronary artery calcification (Agatston score)	[78]
Chen *et al.*	United States	2017	1-4	1541	Male more severe	Scores >100 vs. 0-100 vs. 0, male 65.2% vs. 54.3% vs. 41.7%, $p < 0.001$	Coronary artery calcification (Agatston score)	[41]
He *et al.*	United States	2012	2-4	2018	Male more severe	Score >100 vs. 0-100 vs. 0, male 63.6% vs. 53.3% vs. 41.9%, $p < 0.0001$	Coronary artery calcification (Agatston score)	[60]
Dialysis-dependent CKD								
Lockhart *et al.*	United States	2004	5D (HD)	32	Neutral	Scores high vs. low, male 67% vs. 42%, $p = 0.28$	Abdominal aorta, common iliac, external iliac and common femoral artery calcification on computed tomography	[81]
Liu *et al.*	China	2016	5D	41	Neutral	With vs. without, male 66.67% vs. 59.38%, $p = 0.993$	Immunohistochemical analysis of radial	[80]

							arterial calcium deposition	
Al-Rifai *et al.*	Lebanon	2011	5D (HD)	43	Neutral	No association between VC and gender	Hand X-rays	[28]
Roca-Tey *et al.*	Spain	2009	5D (HD)	45	Neutral	With vs. without, male 81.5% vs. 55.6%, $p = 0.09$	Arteriovenous fistula calcification on computed tomography	[99]
Kim *et al.*	Korea	2019	5D	47	Neutral	With vs. without, male 83.3% vs. 69%, $p = 0.324$	AVF anastomotic segment medial calcification by ultrasound	[72]
Niu *et al.*	China	2019	5D (HD)	56	Neutral	Progression score >500 vs. 100–500 vs. <100, male 70.7% vs. 73.7% vs. 59.3%, $p = 0.572$	Coronary artery calcification (Agatston score)	[92]
Gunen Yilmaz *et al.*	Turkey	2019	5D (HD)	60	Neutral	With vs. without, male 53.3% vs. 53.3%, p = 0.25	Carotid artery calcification on plain radiography and ultrasound	[57]
Lee *et al.*	Taiwan	2019	5D (HD)	61	Neutral	With vs. without, male 52% vs. 37%, p = 0.240	Lateral lumbar radiography (Kauppila score)	[77]
Strózecki *et al.*	Poland	2005	5D (HD)	65	Neutral	With vs. without, male 43.8% vs. 48.5%, $p > 0.05$	Calcified cardiac valves on ultrasound	[103]
Maharem *et al*	Egypt	2013	5, 5D, 5T	73	Neutral	VC presence vs. absence, male 57.9% vs. 31.6%, $p = 0.056$	Pelvic and hand plain radiography	[83]
Petrovic *et al.*	Serbia	2020	5D (HD)	80	Neutral	PWV >8.8 vs. ≤8.8 m/s, male 20% vs. 14%, $p = 0.119$	Pulse wave velocity	[94]
Moldovan *et al.*	Romania	2011	5D (HD)	81	Neutral	With vs. without, male 63.2% vs. 41.7%, $p = 0.44$	Plain radiography of pelvis and hands (Adragao score)	[122]
Jean *et al.*	France	2012	5D (HD)	85	Neutral	Progression vs. stable, male 56% vs. 48%, $p > 0.05$	Multi-site plain radiography involving pelvis, lumbar, knee, right hand, right arm,	[68]

							chest, skull, and orthopantomogram	
Chao *et al.*	Taiwan	2017	5D (HD)	88	Neutral	With vs. without, male 59% vs. 66%, p = 0.54	Aortic arch calcification on chest radiography	[38]
Chandra *et al.*	India	2020	5D	90	Neutral	With vs. without, male 67.5% vs. 62%, p = 0.59	Coronary artery calcification (Agatston score) and thoracic aortic calcification by computed tomography	[35]
Ribeiro *et al.*	Portugal	1998	5D (HD)	92	Neutral	No difference between genders	Calcified cardiac valves on computed tomography	[98]
Chao *et al.*	Taiwan	2020	5D (HD)	96	Neutral	With vs. without, male 47% vs. 43%, p = 0.711	Aortic arch calcification on chest radiography	[37]
Wang *et al*	China	2019	5D (HD)	108	Neutral	Severe vs. mild vs. none, male 30% vs. 23.1% vs. 43.8%, p = 0.08	Abdominal aortic calcification (Kauppila score)	[125]
Avramovski et al.	Macedonia	2019	5D	112	Neutral	No association between VC and gender	Lumbar spine lateral radiography	[30]
Hou *et al*	Taiwan	2019	5D (HD)	120	Neutral	High vs. low, male 52.8% vs. 47.8%, p = 0.851	Pulse wave velocity	[61]
Al Humoud *et al.*	Kuwait	2005	5D	129	Neutral	With vs. without, male 58.8% vs. 45.3%, p = 0.175	Hand X-rays	[62]
Fabbian *et al.*	Italy	2005	5D (HD)	132	Neutral	No difference between genders	Aortic arch calcification on chest radiography	[55]
Bellasi *et al.*	United States	2012	5D (HD)	141	Neutral	Cardiovascular calcification index score 8–11 vs. 5–7 vs. 3–4 vs. 0–2, male 62% vs. 39% vs. 52% vs. 49%, p = 0.57	Lumbar spine lateral radiography (Kaupilla score) and echocardiography-derived valve calcification	[33]

Ballotta *et al.*	Italy	2004	5D	143	Neutral	With vs. without, male 78% vs. 74%, $p = 0.59$	Calcified vessels determined intra-operatively	[32]
Raggi *et al.*	United States	2011	5D (HD)	144	Neutral	Number of calcified valves 2 vs. 1 vs. 0, male 61.1% vs. 44.7% vs. 45.9%, $p = 0.19$	Calcified cardiac valves on computed tomography	[96]
Muntner *et al.*	United States	2007	5D (HD)	148	Neutral	Score ≥ 1000 vs. 400-999 vs. 100-399 vs. 1-99 vs. 0, female 45.5% vs. 46.4% vs. 50% vs. 61.8% vs. 50%, $p = 0.184$	Coronary artery calcification (Agatston score)	[123]
He *et al.*	China	2018	5D (HD)	150	Neutral	With vs. without, male 61.1% vs. 76.2%, $p = 0.099$	Lateral lumbar radiography (Kauppila score)	[59]
Niu *et al.*	China	2019	5D (PD)	150	Neutral	With vs. without, male 51.65% vs. 49.15%, $p = 0.765$	Plain films of lateral abdomen, frontal pelvic, and both hands for abdominal aorta, iliac, femoral, radial, and digital arteries	[91]
London *et al.*	France	2013	5D (HD)	155	Neutral	With vs. without, male-to-female ratio 1.38 vs. 1.44, $p > 0.05$	Common carotid artery calcification by ultrasound	[82]
Kim *et al.*	Korea	2011	5D	184	Neutral	Progression vs. stable, 44% vs. 41%, $p = 0.657$	Aortic arch calcification on chest radiography	[73]
Wu *et al.*	Taiwan	2017	5D (PD)	190	Neutral	Grade 3 vs. 2 vs. 1 vs. 0, male 37.0% vs. 58.3% vs. 40.4% vs. 43.8%, $p = 0.293$	Aortic arch calcification on chest radiography	[109]
Wang *et al.*	Hong Kong	2003	5D (PD)	192	Neutral	With vs. without, male 50.0% vs. 51.5%, $p = 0.842$	Calcified cardiac valves on ultrasound	[108]
Jean *et al*	France	2016	5D (HD)	227	Neutral	Group 3 (severe) vs. 2 vs. 1, male 59.7% vs. 55.6% vs. 59.2%, $p > 0.05$	Lumbar spine lateral radiography	[66]
Chen *et al*	Sweden	2017	5D, 5T	240	Neutral	Score > 100 vs. ≤ 100, male 68% vs. 57%, $p = 0.052$	Coronary artery calcification (Agatston score)	[40]

Chang *et al*	South Korea	2012	5D (HD)	289	Neutral	Severe vs. modest calcification, male 41.8% vs. 44.7%, $p = 0.066$	Lumbar spine lateral radiography	[36]
Floege *et al.*	Multicenter	2010	5D (HD)	360	Neutral	Score ≥ 1000 vs. 400-999 vs. 30-399, 65% vs. 55% vs. 51%	Coronary artery calcification (Agatston score)	[117]
Lee e*t al*	Taiwan	2014	5D (HD)	712	Neutral	Group 3 (severe) vs. 2 vs. 1 vs. none, male 38.1% vs. 42.2% vs. 45.7% vs. 43.8%, $p = 0.606$	Aortic arch calcification on chest radiography	[76]
Ahmed *et al.*	United States	2001	5D	10	Female more common	Male vs. female, 10% vs. 90%, $p < 0.02$	Calciphylaxis on skin biopsy	[26]
Nitta *et al*	Japan	2018	5D (HD)	216	Female more severe	Group 3 (severe) vs. 2 vs. no calcification, female 47.5% vs. 40.9% vs. 23.1%, $p < 0.0001$	Aortic arch calcification on chest radiography	[90]
Komatsu *et al*	Japan	2014	5D (HD)	301	Female common	Grade 2+3 vs. 1 vs. no calcification, male 58.7% vs. 56.3% vs. 77.8%, $p = 0.0009$	Aortic arch calcification on chest radiography	[75]
Maia *et al.*	Brazil	2018	5D (HD)	309	Female more common	With vs. without, male 44.9% vs. 60.8%, $p = 0.039$	Carotid artery calcification on panoramic radiographs	[120]
Disthabanchong *et al.*	Thailand	2018	2-5D, 5T	419	Female more severe (subgroup)	AAC score > 6 vs. ≤ 6 in CKD stage 2 -5, male 44.4% vs. 62.6, p < 0.05<br>In male with stage 5D, 50% vs. 50.5% (P > 0.05)<br>In male with stage 5T, 67.9% vs. 58.5% (p > 0.05)	Lateral lumbar radiography (Kauppila score)	[52]
El Amrani *et al.*	Morocco	2015	5D (HD)	49	Male more common	With vs. without, male 64.7% vs. 26,6 %, $p = 0.014$	Coronary artery calcification (Agatston score)	[53]
Wang *et al.*	China	2014	5D (HD)	77	Male more common	Score >400 vs. 11-400 vs. <10, male 67.8% vs. 53.8% vs. 26.1%, $p < 0.001$	Coronary artery calcification (Agatston score)	[126]
Jankovic *et al*	Serbia	2017	5D (HD)	90	Male more common	With vs. without, male 66.1% vs. 35.3%, $p = 0.008$	Forearm AVF plain radiography	[63]

Jankovic *et al.*	Serbia	2015	5D (HD)	90	Male more severe	Scores 8-11 vs. 4-7 vs. 0-3, 76.7% vs. 50% vs. 38.9%, $p = 0.008$	Plain radiography of pelvis and hands (Adragao score) + forearm AVF plain radiography	[64]
Mazzaferro *et al.*	Italy	2007	5D, 5T	100	Male more severe	Male dialysis vs. male transplant vs. female dialysis vs. female transplant, 1944 vs. 945 vs. 157 vs. 35, $p < 0.02$	Coronary artery calcification (Agatston score)	[84]
Adragao *et al.*	Portugal	2004	5D (HD)	123	Male more severe	Score ≥3 vs. <3, female 22% vs. 58%, $p < 0.001$	Plain radiography of pelvis and hands (Adragao score)	[113]
Coen *et al.*	Italy	2006	5D (HD)	132	Male more severe	Score >1000 vs. 400-1000 vs. 1-400 vs. 0-1, male 74.6% vs. 61.9% vs. 61.3% vs. 38.4% Scores correlated with male gender ($p < 0.05$)	Coronary artery calcification (Agatston score)	[46]
Yoshikawa *et al.*	Japan	2013	5D (HD)	134	Male more common	$\beta = -0.20$, $p = 0.008$	Abdominal aorta calcification on computed tomography	[110]
Kimura *et al.*	Japan	1999	5D (HD)	137	Male more common early during lifetime	With calcification during age 40s, male vs. female 4.0% vs. 3.7%, $p < 0.01$	Abdominal aortic calcification by computed tomography	[74]
Gelev *et al.*	Macedonia	2008	5D (HD)	150	Male more common, especially intimal	VC prevalence: male vs. female, 87.9% vs. 61.0%, $p < 0.03$ Intimal VC: male vs. female, 53.8% vs. 32.2%, $p < 0.02$ Medial VC: male vs. female, 34.1% vs. 28.8%, $p > 0.05$	Pelvic antero-posterior radiography	[118]
Jean *et al*	France	2009	5D (HD)	161	Male more severe	High score (3) vs. no (score 0), male 77% vs. 45%, $p < 0.05$	Multi-site plain radiography involving pelvis, lumbar, knee, right hand, right arm, chest, skull, and orthopantomogram	[67]

Asci *et al.*	Turkey	2010	5D (HD)	207	Male more common and more severe	Score >0 vs. 0, male 57% vs. 41%, $p = 0.05$<br>Score >400 vs. 101-400 vs. 1-100, male 61% vs. 72% vs. 38%, $p = 0.005$	Coronary artery calcification (Agatston score)	[29]
Schlieper *et al*	Serbia	2008	5D (HD)	212	Male more common	With vs. without, male 78% vs. 47%, $p < 0.0001$	Vascular access calcification on plain radiography	[100]
Turan *et al*	Turkey	2016	5D (HD)	224	Male more severe	Group 4 (severe) vs. 3. vs. 2 vs. no calcification, male 56% vs. 59% vs. 38% vs. 41%, $p = 0.003$	Coronary artery calcification (Agatston score)	[106]
Coll *et al.*	Spain	2011	5D	232	Male more common	With vs. without linear calcification, male 65% vs. 41%, $p = 0.01$	Vascular ultrasound for carotid, femoral, or brachial arteries	[47]
Charitaki *et al.*	United Kingdom	2014	5D (HD)	303	Male more severe	Correlation with female, r = -0.124, $p = 0.031$	Pulse wave velocity	[39]
Bae *et al.*	Korea (multicenter)	2016	5D (HD)	423	Male more common and more severe	Male vs. female, positive CAC 69.37% vs. 53.95%, $p = 0.001$<br>Male vs. female, CAC scores 44.1 vs. 5.15, $p = 0.0041$	Coronary artery calcification (Agatston score)	[31]
Renaud *et al.*	France	1988	5D (HD)	24	Male more likely to progress	Correlation coefficient for male vs. annual calcification increase = 1.97, $p < 0.01$	Lumbosacral radiography for linear calcifications involving the abdominal aorta, iliac and femoral arteries	[97]
Tangvoraphonkchai *et al.*	United States	2019	5D (PD)	24	Male more likely to progress	Stable PWV vs. increased PWV, male 33% vs. 75%	Pulse wave velocity	[104]
Alayoud *et al*	France	2020	5D (HD)	28	Male more likely to progress	Progression vs. stable, male 83.3% vs. 36.4%, $p = 0.02$	Coronary artery calcification (Agatston score)	[27]
Choi *et al.*	Korea	2019	5D (HD)	97	Male more likely to progress	Progression vs. stable, male 50.9% vs. 29.5%, $p = 0.033$	Lateral lumbar radiography (Kauppila score)	[43]
Okamoto *et al*	Japan	2018	5D (HD)	184	Male more likely to progress	Annual progression rapid vs. slow, male 53% vs. 27%, $p = 0.008$	Abdominal aorta calcification on	[93]

							computed tomography	
Stage 5T								
Miyatake *et al.*	Japan	2020	5T	50	Neutral	Male vs. female 1.72 vs. 0.00, $p > 0.05$	Abdominal aortic calcification by computed tomography	[86]
DeLoach *et al.*	United States	2009	5T	112	Neutral	With vs. without, male 68.4% vs. 58.1%, $p = 0.29$	Coronary artery calcification (Agatston score) and aortic calcification by computed tomography	[50]
Claes *et al.*	Belgium	2013	5T	115	Neutral	Prevalence male vs. female, 61% vs. 50.7%, $p = 0.16$	Lateral lumbar radiography (Kauppila score)	[45]
Munguia *et al.*	Spain	2015	5T	119	Neutral	With vs. without, 70% vs. 62.3%, $p = 0.384$	Lateral lumbar radiography (Kauppila score)	[89]
Davis *et al.*	United States	2016	5T	131	Neutral	Regression between scores and male gender, r = -0.16~0.051, $p > 0.05$	Iliac arteries calcification by computed tomography	[49]
Keyzer *et al.*	Netherlands	2015	5T	699	Neutral	Tertile 1 (severe) vs. 2 vs. 3, male 58% vs. 55% vs. 57%, $p = 0.73$	T_{50} (propensity of calcification)	[71]
Qureshi *et al*	Sweden	2015	5T	89	Male more severe	Moderate-severe vs. non-minimal, male 76% vs. 54%, $p = 0.04$	Biopsy-verified calcification in epigastric arteries	[95]
Shu *et al*	Taiwan	2012	5T	99	Male more severe	Group 5 (severe) vs. 4 vs. 3 vs. 2 vs. 1, male 66.7% vs. 53.3% vs. 63.6% vs. 65.0 vs. 29.3%, $p = 0.027$	Coronary artery calcification (Agatston score)	[101]
Kahn *et al*	Austria	2017	5T	205	Male more severe, segment-specific	Aorta: male vs. female, 2.0 vs. 1.5, $p = 0.511$<br>Right common iliac artery: male vs. female, 1.0 vs. 1.0, $p = 0.139$	Pelvic computed tomography	[70]

						Total iliac artery: male vs. female, 1.00 vs. 0.50, *p* = 0.003<br>External iliac artery: male vs. female, 1.0 vs. 0.0, *p* <0.001		
Maréchal *et al.*	Belgium	2012	5T	197	Male more likely to progress	Rapid progressor vs. slow progressor vs. non-progressor, male 66% vs. 65% bs. 45%, *p* = 0.02	Coronary artery calcification (Agatston score) and thoracic artery calcification by computed tomography	[121]

AAC, abdominal aortic calcification; CKD, chronic kidney disease; HD, hemodialysis; LAD, left anterior descending; PD, peritoneal dialysis; VC, vascular calcification

Supplementary Table S2. Sex-related risk of vascular calcification in existing studies.

Authors	Country	Time	CKD stages	Sample size	Findings	Results	Calcification assessment method	Ref
Non-dialysis CKD								
Filgueira *et al.*	Brazil	2011	2-4	72	Neutral	Female OR 0.149, $p = 0.82$	Coronary artery calcification (Agatston score)	[132]
Vipattawat *et al.*	Thailand	2014	5, 5D,5T	261	Neutral	For 5T patients, OR 2.49 (0.87–7.14), $p = 0.09$<br>For 5 and 5D patients, OR 2.02 (0.71-5.78), $p = 0.19$	Pelvic and lumbar spine lateral radiography	[153]
Abd Alamir *et al.*	United States	2015	2-3	2070	Neutral	Female OR 1.21 (0.94–1.56)	Mitral annular calcification by coronary computed tomography	[112]
Porter *et al.*	United Kingdom	2007	3-4	112	Male at risk	Male OR 43.713 (2.92–654.0), $p = 0.006$	Coronary artery calcification (Agatston score)	[124]
Stavroulopoulos *et al.*	United Kingdom	2011	3-4	112	Male at risk for progression	Progression at 2-yr, male OR 27.808 (1.625–475.97), $p = 0.022$	Coronary artery calcification (Agatston score)	[149]
Harada *et al.*	Brazil	2014	2-5	117	Male at risk	Male OR 4.92 (2.07–11.70), $p < 0.01$	Coronary artery calcification (Agatston score)	[58]
Chue *et al*	United Kingdom	2012	3	120	Male at risk	Female $\beta = -0.34$ (-13.45– -4.48)	Lumbar spine lateral radiography	[44]
Morena *et al.*	France	2009	1-5	133	Male at risk	Male OR 4.95 (2.36–10.37), $p < 0.0001$	Coronary artery calcification (Agatston score)	[88]
Sigrist *et al*	United Kingdom	2007	4-5D	134	Male at risk for progression	For calcification progression at 2-yr, male OR 8.82 (1.82 to 42.65), $p = 0.007$	Superficial femoral artery calcification by computed tomography	[102]
Sigrist et al.	United Kingdom	2006	4-5D	134	Male at risk	Female $\beta = -2.108$, $p < 0.001$	Superficial femoral artery calcification by computed tomography	[148]
Manghat *et al.*	United Kingdom	2011	1-4	145	Neutral, but male at risk (in subgroup)	Overall, male $\beta = 0.06$, $p =0.54$<br>In those with CKD stage 4, male $\beta = 0.29$, t =2.04, $p =0.049$	Pulse wave velocity based on a stiffness index	[139]
Golembiewska *et al.*	Sweden	2020	5, 5D	149	Male at risk	Male OR 4.4 (1.6–11.1), $p = 0.003$	Inferior epigastric artery histopathology calcification grading	[134]

Dai *et al*	Sweden	2020	5	152	Male at risk	Male OR 6.67 (2.53–17.58)	Histopathology of epigastric artery calcification	[116]
Craver *et al.*	Spain	2013	3-4	178	Male at risk	For AAC severity, male β = 1.237 (0.058-2.417), *p* = 0.04<br>For severe AAC, in all patients, male OR 4.218 (1.403-14.207), p = 0.014<br>For severe AAC, in eGFR < 30: OR 4.167 (1.050-20.178)	Abdominal aortic calcification (Kauppila score)	[48]
Chiu *et al.*	United States	2010	Proteinuric (1-5)	225	Male at risk	Male with significantly higher probability of more severe VC (*p* = 0.01)	Coronary artery calcification (Agatston score)	[42]
Kestenbaum *et al.*	United States	2009	3-5	562	Male at risk but not for progression	For VC presence, incidence rate ratio (IRR) 2.27 (1.26–4.09), *p* = 0.006<br>For VC progression, incidence rate ratio 1.10 (0.84-1.42), *p* = 0.5	Coronary artery calcification (Agatston score)	[119]
Budoff *et al.*	United States	2011	2-3A	1908	Male at risk	Female OR 0.43 (0.35-0.53)	Coronary artery calcification (Agatston score)	[128]
Dialysis-dependent CKD								
Fayed *et al.*	Egypt	2019	5D (HD)	81	Neutral	Male β = 0.088, *p* = 0.695	Abdominal aortic calcification by computed tomography	[131]
Pateinakis *et al.*	Greece	2013	5D (HD)	81	Neutral	β = -0.128, *p* = 0.15	Pulse wave velocity	[144]
Jean *et al.*	France	2012	5D (HD)	85	Neutral	For calcification progression, female OR 0.51 (0.185–1.426), *p* = 0.2	Multi-site plain radiography involving pelvis, lumbar, knee, right hand, right arm, chest, skull, and orthopantomogram	[68]
Wang *et al*	China	2019	5D (HD)	108	Neutral	Female OR 0.56 (0.15–2.06), *p* = 0.38	Abdominal aortic calcification (Kauppila score)	[125]
Cai *et al.*	China	2015	5D (HD)	129	Neutral	Male OR 0.549 (0.113–2.661), *p* = 0.456	Lateral lumbar radiography	[129]
Sumida *et al.*	Japan	2010	5D	135	Neutral	Gender not associated with calcification	Carotid artery calcification by computed tomography	[150]
Gruppen *et al.*	Dutch	2003	5D	140	Neutral	Male *p* > 0.05	Aortic valve calcification on ultrasound	[135]
Sharma *et al.*	United Kingdom	2007	5D	140	Neutral	Female OR 0.45 (0.16–0.81), *p* = 0.53	Mitral annular calcification by cardiac ultrasound	[147]
Muntner *et al.*	United States	2007	5D (HD)	148	Neutral	Prevalence rate ratio 1.37 (0.72–2.62)	Coronary artery calcification (Agatston score)	[123]

Jean *et al*	France	2009	5D (HD)	161	Neutral	Female OR 0.79 (0.3 – 1.8), $p = 0.5$	Multi-site plain radiography involving pelvis, lumbar, knee, right hand, right arm, chest, skull, and orthopantomogram	[67]
Nishizawa *et al.*	Japan	2015	5D (HD)	207	Neutral	Male $\beta = -0.095$, $p = 0.174$	Coronary artery calcification (Agatston score)	[141]
Coll *et al.*	Spain	2011	5D	232	Neutral	Male OR 1.57 (0.69–3.55), $p = 0.27$	Vascular ultrasound for carotid, femoral, or brachial arteries	[47]
Fusaro *et al.*	Italy	2015	5D (HD)	314	Neutral	Male OR 1.52 (0.87–2.66), $p = 0.1$	Lateral lumbar radiography	[133]
Nitta *et al.*	Japan	2018	5D (HD)	216	Female at risk	Female $\beta = 0.137$ (0.021–0.254), $p = 0.0206$	Aortic arch calcification on chest radiography	[90]
Maia *et al.*	Brazil	2018	5D (HD)	309	Female at risk	Female prevalence ratio 2.004 (1.012 – 3.966)	Carotid artery calcification on panoramic radiographs	[120]
Oprisiu *et al.*	France	2002	5D (HD)	24	Male at risk progression	Male gender significant correlation with calcification progression	Pelvic and lumbar lateral radiography	[143]
Jung *et al.*	South Korea	2006	5D (HD)	40	Male at risk for progression	For calcification progression, male $\beta = 1.365$, $p = 0.04$	Coronary artery calcification (Agatston score)	[155]
Nakayama *et al.*	Japan	2013	5D (HD)	47	Male at risk	Female $\beta = -0.407$, $p = 0.014$	Abdominal aortic calcification by computed tomography	[140]
Yamada *et al.*	Japan	2008	5D (HD)	49	Male at risk	Female $\beta = -0.178$, $p = 0.0345$	Digital artery calcification by hand radiography	[154]
Ho et al.	Taiwan	2019	5D	61	Male at risk	Male $\beta = 1688.01$, $p = 0.02$	Coronary artery calcification (Agatston score)	[136]
Wang *et al.*	China	2014	5D (HD)	77	Male at risk	female OR 0.21 (0.07–0.58), $p = 0.003$	Coronary artery calcification (Agatston score)	[126]
Moldovan *et al.*	Romania	2011	5D (HD)	81	Male at risk	Male OR 7.226 (1.138–45.882), $p = 0.036$	Plain radiography of pelvis and hands (Adragao score)	[122]
Jansson *et al.*	Sweden	2019	3-4	84	Neutral for overall risk but male at risk for severe disease	Among total cohort, male not associated with AAC<br>Among those with AAC, male $\beta = 0.413$, $p = 0.03$	Abdominal aortic calcification by computed tomography	[65]
Jankovic *et al.*	Serbia	2017	5D (HD)	90	Male at risk and for severe disease	For VC presence, female OR 0.134 (0.04–0.45), $p = 0.001$	Forearm AVF plain radiography	[63]

						For VC severity, female β = –0.432 (-4.41– -1.86), *p* < 0.001		
Jankovic *et al.*	Serbia	2015	5D (HD)	90	Male at risk for severe disease	Composite calcification score, female β = -0.432, *p* < 0.001	Plain radiography of pelvis and hands (Adragao score) + forearm AVF plain radiography	[64]
Mazzaferro *et al.*	Italy	2007	5D, 5T	100	Male at risk	Male OR 10.5 (3.2–34.4), *p* < 0.0001	Coronary artery calcification (Agatston score)	[84]
Adragao *et al.*	Portugal	2004	5D (HD)	123	Male at risk for severe disease	Score ≥ 3 OR 7.47 (2.9–19.1)	Plain radiography of pelvis and hands (Adragao score)	[113]
Tamei *et al.*	Japan	2011	5D (HD)	127	Male at risk for progression	Progression at 5-yr, male β = 0.969, *p* = 0.0192	Aortic arch calcification by chest radiography	[151]
Bellasi *et al*	United States	2008	5D	142	Male at risk (subgroup)	For coronary artery calcification, male β = 735.82, p = 0.0366<br>For thoracic aorta calcification, gender *p* > 0.05	Coronary artery calcification (Agatston score) and thoracic aorta calcification	[127]
Okamoto *et al.*	Japan	2018	5D (HD)	184	Male at risk for progression	Male OR 3.29 (1.27–8.53), *p* = 0.014	Abdominal aorta calcification by computed tomography	[93]
Turan *et al.*	Turkey	2013	5D (HD)	191	Male at risk	Male RR 2.79 (1.30–5.98), *p* = 0.008	Coronary artery calcification (Agatston score)	[152]
Schlieper *et al.*	Serbia	2009	5D (HD)	194	Male at risk	For composite score, male OR 2.32 (1.19–4.52), *p* = 0.014<br>For Adragao score, male OR 2.75 (1.41–5.38), *p* = 0.003	Plain radiography of pelvis and hands (Adragao score) + forearm AVF plain radiography + Cardiac ultrasound + Carotid ultrasound	[146]
Raggi *et al.*	United States and Europe	2002	5D (HD)	205	Differential effect depending on calcification site	For coronary artery calcification, female β = -0.587547, *p* = 0.0167<br>For aortic calcification, female β = -0.044508, *p* = 0.9036	Coronary artery calcification (Agatston score) and aortic calcification by computed tomography	[145]
Schlieper *et al.*	Serbia	2008	5D (HD)	212	Male at risk	Male OR 5.08 (2.18–11.86), *p* = 0.0001	Vascular access calcification on plain radiography	[100]
Turan *et al.*	Turkey	2016	5D (HD)	224	Male at risk	Male RR 4.14 (2.01–8.51), *p* < 0.001	Coronary artery calcification (Agatston score)	[106]
Chen *et al.*	Sweden	2017	5D, 5T	240	Male at risk	β = 0.35, *p* = 0.008	Coronary artery calcification (Agatston score)	[40]
Nishizawa *et al.*	Japan	2005	5D (HD)	332	Male at risk	Male OR 3.380 (1.289-8.860), *p* = 0.0019	Digital artery calcification by hand radiography	[142]

Floege *et al.*	Multicenter	2010	5D (HD)	360	Male at risk for severe disease	Male β = 0.426, $p = 0.0011$	Coronary artery calcification (Agatston score)	[117]
Ishimura *et al.*	Japan	2002	5D (HD)	421	Male at risk (subgroup)	In diabetics, male OR 3.38 (1.289-8.860), $p = 0.0019$ In non-diabetics, male OR 1.328 (0.252-6.997), $p = 0.7376$	Digital artery calcification by hand radiography	[138]
Ishimura *et al.*	Japan	2004	5D (HD)	594	Male at risk	For aortic calcification, male OR 2.339 (1.466–3.732), $p = 0.0004$ For hand artery calcification, male OR 1.857 (1.043–3.306), $p = 0.0355$	Plain radiography of lateral abdomen and hand	[137]
Stage 5T								
Miyatake *et al.*	Japan	2020	5T	50	Neutral	Female β = -0.051, p = 0.741	Abdominal aortic calcification by computed tomography	[86]
Qureshi *et al.*	Sweden	2015	5T	89	Differential effect depending on calcification site	For epigastric artery, male RR 1.82 (1.03–1.16), $p = 0.03$ For coronary artery, male RR 0.83 (0.38-1.81), $p = 0.63$	Biopsy-verified calcification in epigastric arteries and coronary artery calcification (Agatston score)	[95]
Shu *et al.*	Taiwan	2012	5T	99	Male at risk	Female β = -1.61, $p = 0.0021$	Coronary artery calcification (Agatston score)	[101]
Maréchal *et al.*	Belgium	2012	5T	197	Differential effect depending on calcification site	For coronary calcification, female progression, $p > 0.05$ For thoracic aortic calcification, female progression β = -0.09 (-0.17~-0.01), $p = 0.03$	Coronary artery calcification (Agatston score) and thoracic artery calcification by computed tomography	[121]
Evenpoel *et al.*	Belgium	2015	5T	268	Male at risk	For coronary calcification, female β = -0.64, $p < 0.0001$ For thoracic aortic calcification, female β = -0.32, $p = 0.008$	Coronary artery calcification (Agatston score) and thoracic aortic calcification	[130]

AAC, abdominal aortic calcification; AVF, arteriovenous fistula; CKD, chronic kidney disease; HD, hemodialysis; OR, odds ratio; RR, relative risk; VC, vascular calcification
